# Region-specific reference intervals for TSH in pregnancy: time for changes in Brazil

**DOI:** 10.20945/2359-3997000000067

**Published:** 2018-08-01

**Affiliations:** 1 Faculdade de Medicina de Marília Faculdade de Medicina de Marília Departamento de Medicina Divisão de Endocrinología e Metabolismo Marília SP Brasil Unidade de Tireoide, Divisão de Endocrinología e Metabolismo, Departamento de Medicina, Faculdade de Medicina de Marília, Marília, SP, Brasil

Over the past few years, the diagnosis of thyroid disorders during pregnancy has emerged as one of the major focuses of debate in clinical thyroidology. Whether we should or not universally screening all pregnant women in the first trimester of pregnancy (1) and what is the best pregnancy-trimester specific TSH reference range (2-4) has gained worldwide interest. Answers are necessary to guide clinicians and public health policies.

It is well-known that many complex physiological adaptations in the thyroid economy occur over a normal pregnancy (5). In summary, the increased concentration of hepatic thyroid-binding globulin, of human chorionic gonadotropin, and the elevation of iodide renal clearance, stimulate the maternal thyroid machinery. All together, they lead to marked differences in TSH and thyroid hormone concentrations among pregnant and non-pregnant women, making interpretation of thyroid function tests difficult (6).

Thyroid disorders in pregnancy are relatively frequent, and if not properly treated may be associated with a wide range of maternal and fetal adverse outcomes, most importantly miscarriage, premature delivery, preeclampsia, low fetal weight, reduced cognitive function in offspring and fetal death (7). Thus, despite controversies about the best strategy for screening thyroid dysfunctions in the first trimester of pregnancy, whether should be case-finding or universal screening approach (1), there is an understanding amongst medical societies guidelines that the diagnosis of gestational thyroid diseases should be based on pregnancy-method and region-specific reference ranges (8).

Many centers and countries (including Brazil) do not have own local population-specific TSH reference ranges available. In such cases, previous guidelines (9) recommend using fixed TSH upper limits of 2.5 mU/l or 3.0 mU/l for the first, second or third trimesters, respectively. However, it has been speculated that these reference intervals could result in overdiagnosis, additional investigations and potentially unnecessary levothyroxine treatment for many patients (10). Thererfore, the most recent American Thyroid Association (ATA) guidelines (8) recommend pregnancy-specific and local-specific references ranges for TSH and, if not possible, to adopt a reference interval derived from a population with similar features and TSH assay. If none of this is feasible, the last recommended alternative is to reduce 0.5 mU/l from the reference values for non-pregnant women, which would result in approximately 4.0 mU/l in Brazil.

In fact, reference intervals for laboratory tests are crucial in patient's care by differentiating a healthy individual from the diseased. However, it is a complex task. Pregnancy- and local specific reference limits for TSH can be markedly affected by multiple prenalytical and analytical factors, such as age, gender, ethnicity, antithyroid antibodies, iodine status, TSH assays, and alterations on proteins and free fatty acids levels (11). In addition, the non-Gaussian distribution and a high inter individual variation of TSH levels in the normal population confers an extra challenge (12).

Subgrouping healthy reference intervals by age, gender and other characteristics, may help to improve the diagnostic accuracy (13). The National Academy of Clinical Biochemistry (NACB) has proposed strict selection criteria to establish 95% confident healthy limits for TSH in a population, including absence of family history of thyroid disease, negativity for thyroid antibodies, no visible or palpable goiter, and absence of medications (14).

In this issue of the Archives, Silva de Moraes and cols. report the results of an acclaimed study (15) aiming to establish a local population-specific reference intervals for serum TSH on a Brazilian subpopulation. Using a crosssection design, they included 270 participants aged 18 – 35 years old, with spontaneous pregnancy and gestational age up to 12 weeks, from four prenatal outpatient clinics in a coastal area of the state of Rio de Janeiro.

The study was challenging. Researchers innovated by creating groups with additional and even stricter selection criteria than those of the NCAB to establish TSH reference intervals. In a stepwise approach, they also excluded patients with thyroid ultrasound patterns of thyroiditis and those with iodine insufficiency defined according to the World Health Organization as median urinary iodine concentration < 150 μg/L. A reference group (RG) was composed of 225 participants who filled all NACB criteria. A selective reference group (SRG) was created excluding those with thyroiditis pattern on thyroid ultrasound (n = 170), and at a final step, a more selective reference group (MSRG, n = 130) was defined by excluding any pregnant women with urinary iodine concentration < 150 μg/L.

The TSH reference ranges corresponding to the 2.5^th^ – 97.5^th^ percentile in the first trimester in such studied subpopulation was 0.12 – 4.47 mU/l, 1.26 – 4.0 mU/l and 0.14 – 3.63 mU/l for the RG, SRG and MSRG groups, respectively. These results are in agreement with a previous study in a similar population I from Rio de Janeiro (16) and above the fixed upper limits of 2.5 mU/l recommended in the previous ATA guidelines (9). These data support other recent studies showing that arbitrary cutoffs values for TSH instead of local-specific reference intervals may inappropriately increase the rate of overdiagnosis (17).

Despite some limitation such as the number of patients included being less than 400 (the number required to adequately define reference ranges for measurements with a skewed distribution such as TSH) (18), Silva de Moraes and cols. (15) offer a great contribution to the better understanding and diagnosing of thyroid dysfunction during pregnancy in Brazil.

However, these results cannot be generalized to the entire population. As a matter of fact, in a previous Brazilian study (19) conducted in the continental state of Minas Gerais with 660 pregnant women, the upper limit of serum TSH reference in the first trimester of gestation was 2.68 mU/l. Potential explanations for the divergence include differences in iodine consumption among the populations. A recent trial (20) with pregnant women from Rio de Janeiro showed that this population is iodine sufficient, while some other have reported an increased iodine deficiency prevalence in the state of Minas Gerais (20). In addition, in the study from Minas Gerais (19), thyroid ultrasound and thyroglobulin antibody were not performed to exclude thyroid autoimmunity, but these differences could justify a higher, but not a lower upper serum TSH reference limit.

Taken together, these data suggest that in such a diverse and continental-sized country as Brazil, each region should have its own specific reference values for TSH for each trimester of pregnancy, but this is still far away from reality (21). In addition, these finding may have implications for interpretation of results by clinicians and highlight the need for changes in the current Brazilian guidelines, which still recommends the arbitrary value of 2.5 mU/l for the upper limit of TSH in the first trimester of gestation (22).
